# Habitat-based cetacean density models for the U.S. Atlantic and Gulf of Mexico

**DOI:** 10.1038/srep22615

**Published:** 2016-03-03

**Authors:** Jason J. Roberts, Benjamin D. Best, Laura Mannocci, Ei Fujioka, Patrick N. Halpin, Debra L. Palka, Lance P. Garrison, Keith D. Mullin, Timothy V. N. Cole, Christin B. Khan, William A. McLellan, D. Ann Pabst, Gwen G. Lockhart

**Affiliations:** 1 Marine Geospatial Ecology Laboratory, Nicholas School of the Environment, Duke University, Durham, NC, USA; 2 Bren School of Environmental Sciences and Management, University of California, Santa Barbara, CA, USA; 3 Northeast Fisheries Science Center, National Marine Fisheries Service, Woods Hole, MA, USA; 4 Southeast Fisheries Science Center, National Marine Fisheries Service, Miami, FL, USA; 5 Southeast Fisheries Science Center, National Marine Fisheries Service, Pascagoula, MS, USA; 6 Biology and Marine Biology, University of North Carolina Wilmington, NC, USA; 7 Virginia Aquarium & Marine Science Center, Virginia Beach, VA, USA.

## Abstract

Cetaceans are protected worldwide but vulnerable to incidental harm from an expanding array of human activities at sea. Managing potential hazards to these highly-mobile populations increasingly requires a detailed understanding of their seasonal distributions and habitats. Pursuant to the urgent need for this knowledge for the U.S. Atlantic and Gulf of Mexico, we integrated 23 years of aerial and shipboard cetacean surveys, linked them to environmental covariates obtained from remote sensing and ocean models, and built habitat-based density models for 26 species and 3 multi-species guilds using distance sampling methodology. In the Atlantic, for 11 well-known species, model predictions resembled seasonal movement patterns previously suggested in the literature. For these we produced monthly mean density maps. For lesser-known taxa, and in the Gulf of Mexico, where seasonal movements were less well described, we produced year-round mean density maps. The results revealed high regional differences in small delphinoid densities, confirmed the importance of the continental slope to large delphinoids and of canyons and seamounts to beaked and sperm whales, and quantified seasonal shifts in the densities of migratory baleen whales. The density maps, freely available online, are the first for these regions to be published in the peer-reviewed literature.

The International Whaling Commission placed a moratorium on commercial whaling in 1986, curtailing the biggest direct anthropogenic threat to many cetacean populations. But other threats have persisted, such as bycatch in fisheries^1^, ship strikes^2^, oil spills^3^,^4^, and other pollutants^5^. New threats have been recognized, including naval active sonar^6^,^7^,^8^, other anthropogenic sources of noise^9^,^10^, and climate change^11^. In the United States, national laws protect cetaceans. The Marine Mammal Protection Act (MMPA) prohibits intentional or incidental killing, injuring, or harassment of cetaceans and specifies the circumstances and rules under which permits may be issued for such activities. The Endangered Species Act (ESA) prohibits harm to species threatened with extinction, including 16 cetacean species, and requires conservation of their habitat. The National Environmental Policy Act (NEPA) specifies the process by which U.S. national government agencies must evaluate the potential environmental effects of their actions, consider alternatives, and conduct public reviews. Agency actions that involve decisions to issue permits under the MMPA or ESA are usually subject to this process.

To evaluate the potential effects of proposed activities on cetacean populations, interested parties require a detailed understanding of the spatiotemporal distributions of these populations. Recent developments have created an urgent need for this information in U.S. waters of the Atlantic and Gulf of Mexico, when the U.S. Bureau of Ocean Energy Management (BOEM) proposed to open a large portion of the Atlantic continental shelf to oil and natural gas development and to expand oil and gas leasing in the Gulf of Mexico. Concurrently, the U.S. Navy began development of a new Environmental Impact Statement assessing the effects of training activities proposed for a large portion of the western North Atlantic, while the National Marine Fisheries Service (NMFS) proposed to expand the geographic area designated as critical habitat for endangered North Atlantic right whales, and to reevaluate the status of regional populations of humpback and Bryde’s whales under the ESA.

To estimate the abundance of cetacean species in U.S. waters and assess how they are distributed geographically and seasonally, NMFS and other U.S. government organizations have conducted visual line-transect surveys for over 35 years, yielding two parallel modeling efforts. One effort, prompted by the national regulatory framework, applied distance sampling methodology^12^ to estimate the abundance of cetacean species within large geographic strata^13^,^14^,^15^. The other, driven by a desire to describe cetacean habitats at a fine spatiotemporal scale, developed regression models that related the presence of cetacean species to environmental correlates such as sea surface temperature and then predicted the models across the seascape using gridded maps of the correlates, yielding fine-scale maps of habitat suitability^16^,^17^,^18^.

Neither effort has proved entirely satisfactory for managing cetacean populations in the U.S. The regulatory framework requires an estimate of the number of affected individual animals in proposals for actions that could harm or disturb cetaceans. The broad-scale abundance studies estimated the number of individuals present in large geographic areas but these so-called “stratified models” did not show how they were distributed within each area. In contrast, the habitat suitability studies modeled spatial variability at fine resolutions, but produced estimates that used relative or unit-less scales (e.g. ranging from 0 to 1) that could not directly be used to estimate counts of affected individuals.

The last decade has seen a unification of these two approaches into a two-stage method known as density surface modeling^19^,^20^, in which traditional distance sampling is coupled with multivariate regression modeling to produce density maps (individuals km^−2^) predicted from fine scale environmental covariates^21^. A challenge with density surface models (DSMs) is that a large number of sightings are needed to fit the regression model. Cetaceans are rare; often many surveys must be aggregated to obtain sufficient sightings. For example, a study of beaked whales in the eastern tropical Pacific aggregated 6 years of surveys to obtain just 90 sightings of Cuvier’s beaked whale and 106 of *Mesoplodon* beaked whales^22^. This problem is exacerbated if the modeler desires to fit different models for different regions or seasons under the presumption that different behaviors occur in those places and times, e.g. that baleen whales on summer feeding grounds exhibit different environmental preferences than those on winter calving grounds.

Here, we present cetacean density models for the U.S. Atlantic and Gulf of Mexico. To maximize the number of taxa modeled and account for regional and seasonal variability, we aggregated nearly 1.1 million linear km of line-transect surveys conducted over 23 years by 5 institutions. We modeled density from 8 physiographic and 16 dynamic oceanographic and biological covariates, producing predicted density maps for 29 cetacean species and multi-species guilds, comprising 36 species in total. Results are freely available online at the Ocean Biogeographic Information System Spatial Ecological Analysis of Megavertebrate Populations (OBIS-SEAMAP) repository at http://seamap.env.duke.edu/models/Duke-EC-GOM-2015.

## Results

**Figure 1:**
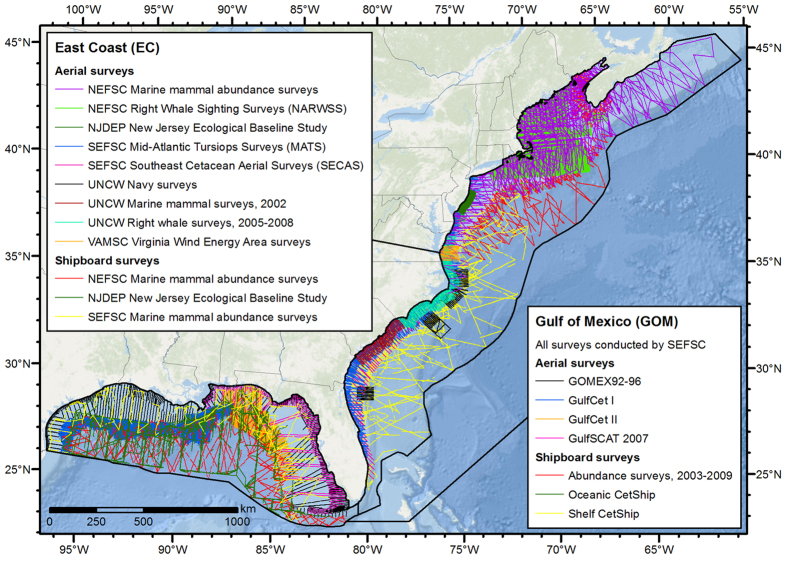
Analysis regions and line transect surveys used in this study. See Table 1 for more survey details. Surveyors: NOAA NMFS Northeast Fisheries Science Center (NEFSC), New Jersey Department of Environmental Protection (NJDEP), NOAA NMFS Southeast Fisheries Science Center (SEFSC), University of North Carolina Wilmington (UNCW), Virginia Aquarium & Marine Science Center (VAMSC). Figure produced with ArcGIS 10.2.2 (http://www.arcgis.com); background map credits: Esri, DeLorme, GEBCO, NOAA NGDC, and other contributors.

The surveys contributed by the collaborators spanned the U.S. Exclusive Economic Zone (EEZ) of the Gulf of Mexico and eastern U.S., as well as a portion of Canada’s EEZ (Fig. 1, Table 1). The Gulf of Mexico is partially isolated from the North Atlantic and exhibits distinct biogography. To allow for the possibility that species-environment relationships differ between the Gulf of Mexico and western North Atlantic, we split our study area at 80.5°W into two analysis regions, the Gulf of Mexico (GOM) and East Coast (EC) and fitted density models to them separately.

To summarize the results, we grouped the cetacean species reported by the surveys into four taxonomic groups according to their phylogeny and ecology (Table 2, Supplementary Table S1): small delphinoids (i.e., small species of superfamily *Delphinoidea*), comprising harbor porpoise (*Phocoena phocoena*) and 11 dolphin species (genera *Delphinus*, *Lagenodelphis*, *Lagenorhynchus*, *Stenella*, *Steno*, and *Tursiops*); large delphinoids, comprising 7 species (genera *Feresa*, *Globicephala*, *Grampus*, *Orcinus*, *Peponocephala*, and *Pseudorca*); beaked and sperm whales, comprising 3 sperm whale species (genera *Kogia* and *Physeter*) and at least 6 beaked whale species (genera *Hyperoodon*, *Mesoplodon*, and *Ziphius*); and baleen whales, comprising 7 species (genera *Balaenoptera*, *Eubalaena*, and *Megaptera*).

In total, we incorporated 26,307 sightings into the analysis. Most small delphinoid and baleen whale sightings reported complete taxonomic identifications. A smaller number reported an ambiguous identification of two possible species (e.g. “fin or sei whale”) that we classified into one or the other using habitat variables (see Methods). In contrast, many sightings of the other two taxonomic groups reported ambiguous identifications, mainly owing to the difficulty of visually distinguishing species of pilot whales (*Globicephala melas* and *G. macrorhynchus*), beaked whales (*Mesoplodon spp.* and *Ziphius cavirostris*), and dwarf and pygmy sperm whales (*Kogia sima* and *K. breviceps*) from a distance. Lacking sufficient fully-resolved sightings to attempt a classification of these ambiguous sightings from habitat variables, we modeled these as three multi-species guilds (Supplementary Table S1). Finally, 5371 sightings were too taxonomically ambiguous to be incorporated into the analysis (Supplementary Table S2), resulting in an underestimation of density and abundance, due to some animals being sighted but not accounted for.

In the GOM, we modeled the density of 17 species and 2 multi-species guilds—beaked whales and *Kogia*. We fitted DSMs for all but 3 infrequently-sighted taxa; for these, we produced traditional stratified models that estimated mean density over the area they were likely to inhabit (see Methods). In the EC, we modeled 25 species and 3 guilds—beaked whales, *Kogia*, and pilot whales. Of these 28 modeled taxa, we fitted DSMs for 15 and stratified models for 13. The EC study area, spanning more than 20° of latitude, covered portions of two marine ecoregions—a northern, cold, productive region separated at roughly 35° N by the Gulf Stream Current from a southern, warm, less-productive region^23^. By spanning more habitats, the EC yielded a higher species richness than the GOM, but because it only covered the extremes of some species’ ranges, some species were sighted rarely and could not be modeled with DSMs.

We now turn to the results for individual models. It is not possible to give full treatment to each here; we discuss the four taxonomic groups instead. For each group, we present an aggregate density surface that sums the mean density surfaces of the taxa making up the group, then discuss patterns in distribution and highlight important results for individual taxa. The individual model results are available at the OBIS-SEAMAP repository (http://seamap.env.duke.edu/models/Duke-EC-GOM-2015), including density and uncertainty surfaces and summary reports that describe important taxon-specific modeling decisions and parameter estimates (e.g. *g*(0)) and provide diagnostic maps, plots, and statistical output.

### Small delphinoids

**Figure 2:**
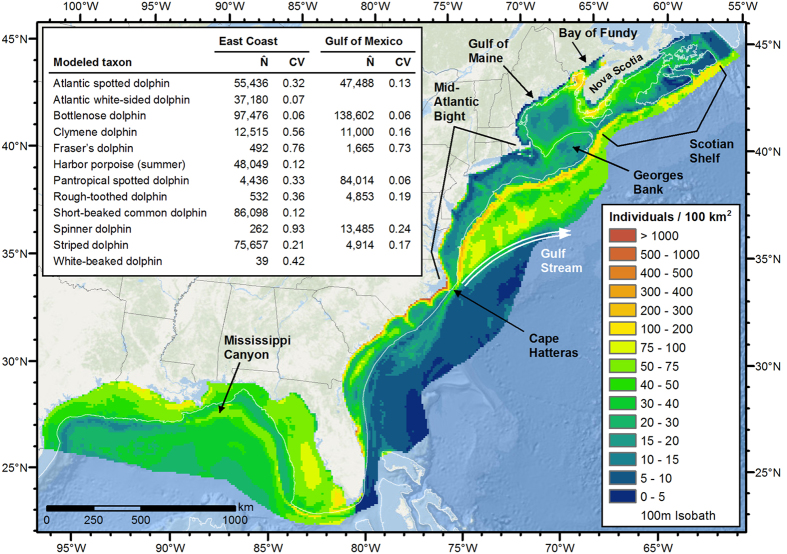
Predicted mean density of small delphinoids. The inset table lists the estimated mean abundance (number of individuals, N̂) and associated coefficient of variation (CV) for each taxon. The estimates are the year-round mean except for harbor porpoise. Harbor porpoise was modeled with two seasonal models instead of a year-round model; the estimates listed are for the summer model, defined as June–October for this species. Figure produced with ArcGIS 10.2.2 (http://www.arcgis.com); background map credits: Esri, DeLorme, GEBCO, NOAA NGDC, and other contributors.

Fewer small delphinoid species were sighted in the GOM than the EC. In aggregate, the models predicted a more uniform spatial distribution in the GOM and a more heterogeneous distribution in the EC (Fig. 2).

In the GOM, all species but two exhibited spatial distributions distinct from the others, consistent with prior suggestions of habitat partitioning among Gulf of Mexico odontocetes^24^,^25^. In neritic waters, bottlenose dolphins dominated nearshore areas, while Atlantic spotted dolphins dominated the mid and outer shelf. In oceanic waters, Clymene dolphins concentrated in the western and central GOM, pantropical spotted dolphins in the east and center, striped dolphins in the northeast near the Mississippi Canyon, and spinner dolphins in moderately deep slope waters of the central and eastern GOM. Rough-toothed dolphins occurred on and off the shelf with no dominant spatial pattern; our model showed a slight association with high-slope bathymetry. Fraser’s dolphins were sighted too infrequently to model with a DSM, but all sightings occurred off the shelf.

In the EC, four distinct zones emerged in the aggregate distribution of small delphinoids. In the first, the warm, neritic waters south of Cape Hatteras, bottlenose and Atlantic spotted dolphins dominated, similar to the GOM shelf, except that Atlantic spotted dolphins were concentrated only in the mid-shelf region. Close to the shelf break, they were displaced by the “offshore” ecotype of bottlenose dolphins^26^. Throughout the shelf, several other species were also present, but in densities too low to model with DSMs.

The second zone, the neritic waters north of Cape Hatteras, is characterized by colder, nutrient-rich waters that exhibit high seasonal productivity. In the Mid-Atlantic Bight, our models predicted seasonal changes in species composition, however survey effort was limited in winter so these predictions must be viewed cautiously. In winter, the models predicted that harbor porpoises and Atlantic white-sided dolphins occupied the mid- and outer shelf, while in summer, these species moved north, giving way to bottlenose dolphins, which are known to migrate northward in summer^27^, and Atlantic spotted dolphins. Short-beaked common dolphins were predicted to occupy the mid- and outer shelf. To the north—Georges Bank, the Gulf of Maine, Bay of Fundy, and Scotian Shelf—harbor porpoises and Atlantic white-sided and short-beaked common dolphins dominated, with lesser numbers of bottlenose dolphins and very low densities of white-beaked dolphins. Consistent with prior reports^28^, harbor porpoises were predicted to aggregate in summer in the northern Gulf of Maine and lower Bay of Fundy and, to a lesser degree, off the southern tip of Nova Scotia.

The third zone, the oceanic waters of the Gulf Stream and southward, is consistently warm and nutrient-poor relative to the other zones. This area received relatively little survey effort except in specific areas along the shelf break (Fig. 1, UNCW Navy surveys), and our models’ predictions should be viewed cautiously. Although species richness was high in this zone, aggregate density was lowest among the four zones. Bottlenose and Atlantic spotted dolphins were the most abundant species; this contrasts with the GOM, where their off-shelf density was very low.

The fourth zone, the oceanic waters north of the Gulf Stream, is cold and nutrient-rich. Although this area was predicted to sustain the highest aggregate density of dolphins of the four EC zones, almost all surveys here occurred in summer; caution must be exercised when utilizing our predictions in non-summer months. Highest densities occurred at the shelf break and along the continental slope, with bottlenose and short-beaked common dolphins concentrating here, and Atlantic white-sided dolphins to a lesser degree. Far from the shelf, striped, bottlenose, and Atlantic spotted dolphins dominated.

Finally, where the EC and GOM models met, at 80.5°W, an area of very low survey effort, the aggregate distribution of small delphinoids showed a clear discontinuity, with very low density predicted by the EC models and moderate density predicted by the GOM models. It may be possible to address this problem in a future revision of our models by including additional survey data or defining a new study area that spans both the Gulf of Mexico and southeast continental U.S.

### Large delphinoids

**Figure 3:**
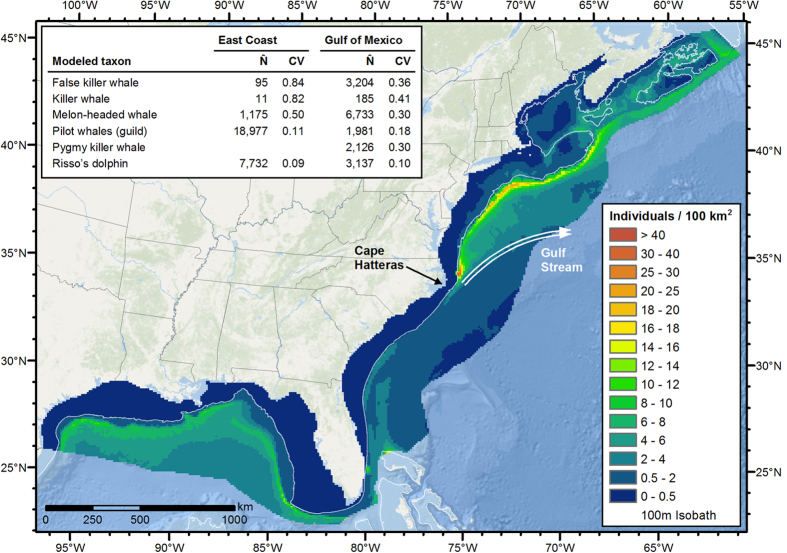
Predicted mean density of large delphinoids. The inset table lists the estimated mean abundance (number of individuals, N̂) and associated coefficient of variation (CV) for each taxon. In the East Coast region, pilot whales comprised a guild of two species; in the Gulf of Mexico, only one pilot whale species was present. Figure produced with ArcGIS 10.2.2 (http://www.arcgis.com); background map credits: Esri, DeLorme, GEBCO, NOAA NGDC, and other contributors.

Large delphinoids occurred in the GOM exclusively in oceanic waters (Fig. 3). Here, we modeled six species. Killer, false killer, and pygmy killer whales exhibited relatively uniform spatial distributions. Melon-headed whales were slightly less uniform, with higher abundance to the north. Risso’s dolphins and short-finned pilot whales concentrated along the continental slope.

In the EC, we modeled five individual species and one guild, pilot whales. Pilot whales and Risso’s dolphins predominated and were the only taxa sighted frequently enough to model with DSMs. Both were predicted to occur throughout oceanic waters, in highest density along the continental slope, consistent with prior reports^29^,^30^. Pilot whales were especially concentrated off Cape Hatteras, just north of where the Gulf Stream separates from the shelf. Both were also predicted in lower density over the shelf in northern, cold, productive waters. We modeled the remaining four species with stratified models. Killer and false killer whales were sighted and assumed to occur both on and off the shelf, while melon-headed and northern bottlenose whales were sighted and assumed to occur only in oceanic waters.

### Beaked and sperm whales

**Figure 4:**
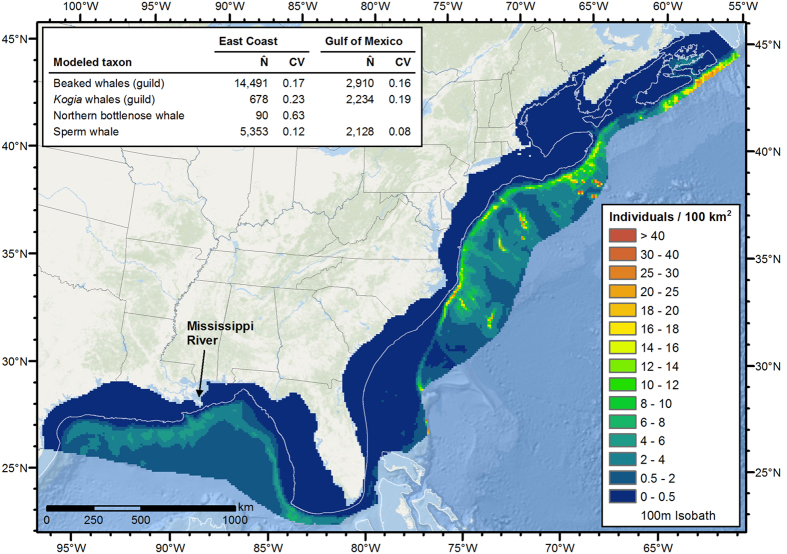
Predicted mean density of beaked and sperm whales. The inset table lists the estimated mean abundance (number of individuals, N̂) and associated coefficient of variation (CV) for each taxon. Figure produced with ArcGIS 10.2.2 (http://www.arcgis.com); background map credits: Esri, DeLorme, GEBCO, NOAA NGDC, and other contributors.

For beaked and sperm whales, deep-diving teuthivores, our models predicted patchy distributions concentrated in deep waters over high-relief bathymetry, in keeping with evidence of high prey density in these areas^31^. In the GOM, models predicted concentrations near off-shelf submarine canyons at the mouth of the Mississippi River and the central northern Gulf^32^, and along the continental slope (Fig. 4). In the EC, the models predicted highest densities along the continental slope, in and around submarine canyons, and near seamounts, particularly in cold, productive waters, consistent with their reported habitats^16^,^33^. The models also predicted sperm whales in northern shelf waters, but in much lower densities.

### Baleen whales

Baleen whales were sighted mainly in the EC, where observers reported seven species. Blue whales and Bryde’s whales were sighted very infrequently; we modeled them with stratified models. We modeled the five remaining species—fin, humpback, minke, sei, and North Atlantic right whales—with DSMs. Most baleen species undertake large seasonal migrations, moving into cold, productive waters in summer to feed and travelling to warmer, calmer waters in winter to calve or breed. Under the assumption they would express different habitat preferences during different times of the year, we split the survey data into seasonal strata with species-specific monthly ranges and fitted separate DSMs to each. For humpback and minke whales, we used two seasons; for right and sei whales, we used four. For fin whales, which remain in the area during winter in substantial numbers^34^, we used a year-round model.

**Figure 5:**
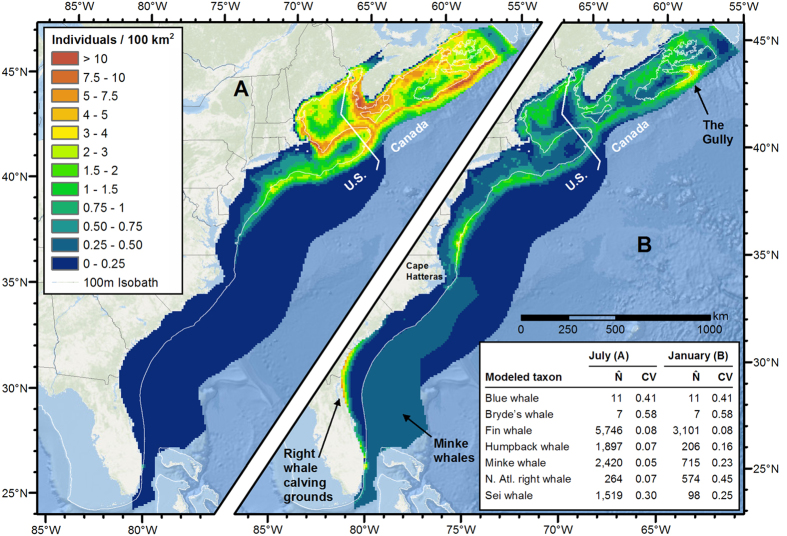
Predicted mean density of baleen whales in July (A) and January (B) for the East Coast region. The inset table lists the predicted mean monthly abundance (number of individuals, N̂) and associated coefficient of variation (CV) for each taxon. Figure produced with ArcGIS 10.2.2 (http://www.arcgis.com); background map credits: Esri, DeLorme, GEBCO, NOAA NGDC, and other contributors.

We predicted all baleen whale models at a monthly time step; all showed plausible temporal patterns in density that were consistent with the literature. To summarize the strong temporal dynamics, we present aggregate density maps for two months, July and January, representative of summer and winter. In July (Fig. 5A), the models predicted that baleen species aggregate in productive northern waters, concentrated near the continental shelf break and near on-shelf areas of high bathymetric relief, such as the edges of banks and ledges. Fin whales were most abundant, followed by minke, humpback, sei, and right whales.

In January (Fig. 5B), north of Cape Hatteras, density of all five species was predicted to be much lower, as most individuals were presumed to have migrated out of the study area to calving grounds, while some remained to overwinter, consistent with reports of lower but non-zero wintertime abundance of these species^34^,^35^,^36^,^37^. We lacked sufficient survey effort in Canadian waters to offer predictions for this area in winter models of humpback, minke, right, and sei whales. This situation did not apply to the year-round model fitted for fin whales. This model predicted fin whales in Canadian waters throughout the year in areas of high relief, especially in the vicinity of the Gully, a submarine canyon at the outer edge of the Scotian Shelf. While fin whales consistently inhabit the Gully in summer^38^, their status in winter is unknown, although fin whales have been reported in December in other northern areas, including western Greenland and the Gulf of St. Lawrence^39^,^40^. South of Cape Hatteras, our winter models predicted North Atlantic right whales in high density in their near-shore calving grounds, and other species present in broader areas in lower densities. The winter minke whale model predicted moderate density off the shelf, consistent with acoustic monitoring results and the hypothesis that the area is a minke whale calving ground^41^.

In the GOM, surveys reported on-effort sightings of only two baleen whale species. A single fin whale was sighted in the western Gulf at the shelf break. Fin whales do not inhabit the northern Gulf of Mexico, but our process was to model all species reported while observers were on effort, regardless of rarity; accordingly, we incorporated this extralimital sighting into a GOM-wide stratified model. For the other species, Bryde’s whale, we fitted a limited DSM designed to reflect their distribution along the northeast Gulf slope.

## Discussion

The goal of this effort was to develop the most comprehensive and detailed models of cetacean density possible for our study regions. To this end, we aggregated a very large collection of survey data (23 years) to maximize our coverage of cetacean species, seasons, and geographic areas. We then applied a modeling strategy that scaled the spatial, temporal, and taxonomical resolution of models according to how frequently taxa were sighted, how easy they were to identify, and what was known of their ecology. At one extreme—species that were sighted frequently, easy for observers to identify, and well known in the literature, such as the most common baleen whales—our strategy was to fit species-specific DSMs with seasonal and sub-regional strata, allowing seasonal and regional differences in species-environment relationships to be modeled explicitly. When our data suggested seasonal movements and the literature concurred, we made predictions at monthly resolution, to reproduce temporal shifts in density as species migrate. At the other extreme—species that were rarely sighted, hard to identify, and poorly known, such as *Kogia* in the EC region—our strategy was to revert to traditional stratified models, model multiple species together as a guild, or both, reflecting the relative scarcity of information. When reasonable, we substituted data from other regions to compensate, e.g. by drawing upon sightings from external surveys to improve fits of detection functions, or utilizing estimates of availability and perception bias from external studies when none were available from studies in our region. For the middle ground—species that were sighted at modest rates and moderately well known, such as many of the oceanic odontocetes—our strategy was to fit single-season DSMs and provide year-round average predictions, reflecting a modest confidence in what was known of these species’ distributions. This scaling of methodology to available data and knowledge made our modeling strategy adaptable to the variety of distributions and abundances of cetacean species found in the U.S Atlantic and Gulf of Mexico.

In total, we developed DSMs for 84% of the taxa sighted in the GOM and 54% sighted in the EC, and produced monthly predictions for 11 taxa (Supplementary Table S3). This represents an increase over prior habitat-based cetacean modeling efforts^17^,^42^,^43^,^44^ both in the number of taxa modeled and the spatial extent and temporal resolution of predictions. The availability of these more taxonomically and spatiotemporally comprehensive results, at finer temporal resolution, is especially useful for strategic marine spatial planning of human activities that are potentially harmful to cetaceans, particularly for endangered migratory species. However, it is important to bear in mind that in the U.S. the MMPA requires that cetaceans be managed on a per-stock basis (the statute defines a “stock” to be “a group of marine mammals of the same species or smaller taxa in a common spatial arrangement that interbreed when mature”) and that NMFS recommends that data older than 8 years not be used to estimate stock-level parameters required by the statute such as the Minimum Population Estimate (N_min_) or Potential Biological Removal (PBR)^45^. Thus our results may not be directly suitable for estimating these parameters, especially for species that exhibit complex stock structure within our study area, such as bottlenose dolphins. Nevertheless, the NMFS stock assessment process does not produce spatiotemporally-explicit descriptions of cetacean stocks (i.e. density maps), and our results remain the best available alternative for spatiotemporally-explicit management problems, such as those that require estimates of potential cetacean “takes” resulting from proposed human activities.

In addition to providing unprecedented taxonomical coverage and temporal resolution, we were able to develop models that considered a wider range of dynamic oceanographic features than have previously been incorporated into cetacean density models (Supplementary Table S3). By including covariates related to meso-scale fronts and eddies, as well as several formulations of biological productivity, we sought both to improve the explanatory and predictive power of our models and to test the importance of these predictors relative to more commonly-used predictors such as bathymetry and sea surface temperature (SST), thereby contributing to the understanding of these species’ spatial ecology. Although these results are not the focus of this paper, we discuss some of them in the species-specific reports that are available online with the predicted density surfaces through the OBIS-SEAMAP repository (http://seamap.env.duke.edu/models/Duke-EC-GOM-2015).

Our models provide a new baseline for understanding the ecological relationships and conservation status of cetaceans in the western North Atlantic and Gulf of Mexico. We plan to regularly update these models as new survey data become available, such as the NOAA Atlantic Marine Assessment Program for Protected Species (AMAPPS) surveys and continuing UNCW and NARWSS surveys. Extending the temporal extent of surveys used in our modeling efforts raises important issues concerning the appropriate range of time to be included in the development of models. As the data collection period is increased, changes in population demographics, environmental changes, or climatic trends may be inappropriately incorporated into model development. For example, significant changes in population demographics such as increases in the population of North Atlantic right whales^46^, or changes to species distributions following environmental disturbances such as the *Deepwater Horizon* oil spill, could be masked or exaggerated if temporal trends are not carefully considered. To accomplish this goal, we plan to conduct independent comparative tests of future model revisions to ascertain when past survey data should be removed from the collection when they are no longer representative of current trends and distribution patterns, and explore the possibility of weighting more recent survey data during the model fitting stage.

## Methods

### Surveys and study area

An overriding goal of our study was to maximize the number of cetacean species modeled with DSMs rather than stratified models. Meeting this goal required many sightings, thus many surveys. We established collaborations with 5 institutions that collectively conducted nearly 1.1 million linear km of line-transect surveys for marine mammals collected in our area of interest under appropriate U.S. federal permits, spanning the years 1992–2014. (Fig. 1, Table 1). We only considered surveys that used two or more observers and adhered to the requirements of distance sampling methodology^12^. We acquired the original survey data files, aggregated them into a common spatial database, and manually delineated a study area encompassing the total area surveyed (Fig. 1). To allow for the possibility that species-environment relationships differ between the Gulf of Mexico and western North Atlantic, we split our study area at 80.5°W into two analysis regions, the Gulf of Mexico (GOM) and East Coast (EC).

### Modeled taxa

To facilitate straightforward use of our results within in the U.S. regulatory framework, we sought to model density on a per-species basis. This required that all cetacean sightings have fully-resolved taxonomic identifications, but some species were difficult for observers to discern in the field, resulting in a nontrivial fraction of sightings that were not fully resolved taxonomically (Table 2).

We handled these ambiguous sightings differently based on their degree of ambiguity. The least ambiguous sightings resolved the taxonomic identification to a pair of species, *e.g.* “fin or sei whale”. When there were a substantial number of these for a pair of species, plus a substantial number of fully-resolved sightings for both, and the literature or exploratory analysis suggested the two exhibit different spatiotemporal distributions, we classified the ambiguous sightings into one species or the other using the random forest classifier *cforest*^47^ from the R *party* package version 1.0-23. The classifier was trained on the fully-resolved sightings, using the species identification as the response variable, and environmental variables (Supplementary Table S3), day of year, or group size as predictor variables, depending on the species. The default parameters for *cforest* were used, with 1000 trees. Receiver operating characteristic (ROC) curve analysis yielded a threshold for classifying sightings into one species or the other; we selected the value that maximized the Youden index.

When we lacked enough fully-resolved sightings to build a classifier, or could not establish a plausible claim that the two species exhibited sufficiently different spatiotemporal distributions, we modeled the two species together as a guild that included both the ambiguous and the fully-resolved sightings of both species. This occurred for the *Kogia* (dwarf and pygmy sperm whales) in both analysis regions and the *Globicephala* (short-finned and long-finned pilot whales) in the EC region.

The next most ambiguous type of sightings resolved the identification to a genus or family of more than two species. This occurred for the *Ziphiidae* family (beaked whales), for which the number of “*Mesoplodon* species” or “*Ziphiidae* species” sightings dominated the number of fully-resolved sightings. We modeled all these as a single “beaked whales” guild.

Finally, the most ambiguous sightings indicated only that a “dolphin” or “whale” was sighted, often with a size qualifier, e.g. “large whale”. We omitted these sightings from our analysis. Although these sightings were a clear minority compared to the fully-resolved sightings, they resulted in an underestimation of density on account of animals being present and sighted but not included in a model.

### Density surface modeling

After preparing each taxon’s sightings for modeling, we followed the two-stage density surface modeling approach described by Miller *et al.*^20^, using covariates associated at the observation level. In the first stage, we fitted detection functions that modeled the detectability of the taxon according to distance from the trackline and other covariates^12^. We used a distance sampling approach that relied on a single observer team; over 80% of our surveys used a single team, precluding use of dual-team methods such as mark-recapture distance sampling^48^. For surveys that used two teams, we utilized sightings only from the primary team. To obtain sufficient sightings for the detection functions while accounting for survey-specific influences on detectability, we organized the surveys into a hierarchy according to their platform and protocol similarity and fitted detection functions to groups of surveys within the hierarchy (Supplementary Figs S1, S2). Then we split the survey transects into segments and applied the detection functions to estimate abundance for each segment using a Horvitz Thompson-like estimator^19^. To correct for availability and perception biases, we applied estimates of the *g*(0) parameter^12^ reported by the original surveys, or if none were reported, obtained from the literature.

In the second stage, we fitted generalized additive models (GAMs)^49^ that modeled per-segment abundance from spatially- and temporally-varying environmental covariates believed to correlate with cetacean distributions, including physiographic, physical oceanographic, and biological covariates (Supplementary Table S3). Physiographic covariates included depth, slope, and distance to shore, canyons, seamounts, and ecologically relevant isobaths. Physical oceanographic covariates included SST, distance to SST fronts, wind speed, total and eddy kinetic energies, and distance to geostrophic eddies derived from sea surface height observations made by satellites. Biological covariates included chlorophyll concentration observed by satellite, primary production, and potential biomass and production of zooplankton and epipelagic micronekton obtained from a bio-physical ocean model.

Not all temporally-varying covariates were available across the entire time range spanned by the 1992–2014 study period. For example, all of the biological covariates were derived in part from satellite ocean color observations that first became available in late 1997. To address this problem without simply discarding cetacean surveys due to missing covariate values, we tested a series of GAM formulations for each model. We started with just the physiographic covariates, which resulted in no data loss, then added the physical oceanographic covariates, dropping surveys conducted outside 1993–2013 when using covariates derived from satellite altimeters, and finally added the biological covariates, dropping surveys conducted prior to late 1997. To allow for the possibility that different species correlate with temporally-varying covariates at different time scales, we tested both 8-day climatological estimates of covariates (e.g. mean SST for a given 8 days of the year, averaged over 30 years), which reflected only regular seasonal variability, and contemporaneous estimates (e.g. SST on the date of the survey segment, from a daily satellite image), which reflected inter-annual and ephemeral variations, in addition to regular seasonal changes. After examining model performance statistics and other criteria, we selected one model as “best”; we describe the details of this procedure in Supplementary Information.

When the literature suggested a taxon behaves differently during different times of the year or in different parts of the study area, as with baleen whales^50^, we separated the segments into seasonal or sub-regional strata and fitted a separate series of GAMs to each; otherwise we fitted a single series of GAMs to all segments in the analysis region. After selecting the best model for each stratum, we predicted it across the modeled region and season using gridded maps of the covariates, obtaining density surfaces for each taxon, with associated estimates of uncertainty. In response to specific requests voiced by prospective model users, including NMFS and the U.S. Navy, and reflecting the spatial resolution of environmental covariates, we provided predictions at 10 km resolution. When insufficient sightings of a taxon were available to utilize covariates, we fitted a model without them, resulting in a so-called stratified model that produced a mean density estimate for the modeled region. Our uncertainty estimates reflect the uncertainty in the GAM parameter estimates (or mean density estimate, for stratified models), but do not include uncertainty in detection functions or availability or perception bias estimates, and therefore underestimate actual uncertainty (see Supplementary Information for further discussion).

A detailed description of both stages of the density surface modeling procedure is given as Supplementary Information online.

## Additional Information

**How to cite this article**: Roberts, J. J. *et al.* Habitat-based cetacean density models for the U.S. Atlantic and Gulf of Mexico. *Sci. Rep.* **6**, 22615; doi: 10.1038/srep22615 (2016).

## Supplementary Material

Supplementary Information

## Figures and Tables

**Table 1:** Line transect surveys used in this analysis.

Region	Platform	Surveyor	Survey program	Years	Length (1000 km)	Hours
EC	Aerial	NEFSC	Marine mammal abundance surveys^15^	1995–2008	70	412
			Right Whale Sighting Survey (NARWSS)^51^	1999–2013	432	2330
			NARWSS harbor porpoise survey^51^	1999	6	36
		NJDEP	New Jersey Ecological Baseline Study^52^,^53^	2008–2009	11	60
		SEFSC	Mid–Atlantic Tursiops Surveys (MATS)	1995, 2004–5	35	196
			Southeast Cetacean Aerial Surveys (SECAS)^54^	1992, 1995	8	42
		UNCW	Cape Hatteras Navy surveys^55^	2011–2013	19	125
			Jacksonville Navy surveys^55^	2009–2013	66	402
			Marine mammal surveys, 2002^26^	2002	18	98
			Onslow Bay Navy surveys^55^	2007–2011	49	282
			Right whale surveys, 2005–2008^55^	2005–2008	114	586
		VAMSC	Virginia Wind Energy Area surveys^56^	2012–2014	9	53
			Total:	1992–2014	837	4622
	Shipboard	NEFSC	Marine mammal abundance surveys^15^	1995–2004	16	1143
		NJDEP	New Jersey Ecological Baseline Study^52^,^53^	2008–2009	14	836
		SEFSC	Marine mammal abundance surveys^57^	1992–2005	28	1731
			Total:	1992–2009	58	3710
GOM	Aerial	SEFSC	GOMEX92–96^54^	1992–1996	27	152
			GulfCet I^58^	1992–1994	50	257
			GulfCet II^59^	1996–1998	22	124
			GulfSCAT 2007	2007	18	95
			Total:	1992–2007	117	628
	Shipboard	SEFSC	Oceanic CetShip^14^	1992–2001	49	3102
			Shelf CetShip^13^	1994–2001	10	707
			Marine mammal abundance surveys^60^	2003–2009	19	1156
			Total:	1992–2009	78	4965

Length and hours are the cumulative linear distance and duration observers were on effort for each survey program (references given). See Fig. 1 for spatial effort. Surveyors: NOAA NMFS Northeast Fisheries Science Center (NEFSC), New Jersey Department of Environmental Protection (NJDEP), NOAA NMFS Southeast Fisheries Science Center (SEFSC), University of North Carolina Wilmington (UNCW), Virginia Aquarium & Marine Science Center (VAMSC).

**Table 2:** Sightings reported and taxa modeled.

Region	Taxonomic group	Sightings retained for analysis		Taxa modeled with	
		Fully-resolved	Ambiguous	DSMs	Stratified models
EC	Small delphinoids	10274	944	6	6
	Large delphinoids	817	823	2	3
	Beaked and sperm whales	581	181	2	2
	Baleen whales	7680	646	5	2
GOM	Small delphinoids	3061	151	7	1
	Large delphinoids	410	13	5	1
	Beaked and sperm whales	442	258	3	
	Baleen whales	18	8	1	1

Fully-resolved sightings had a complete taxonomic identification. Ambiguous sightings that were retained for analysis were classified into one of the 29 modeled taxa (see Methods). Taxa modeled with stratified models were sighted so infrequently that a DSM could not be fitted; instead, we produced traditional mean density estimates for the geographic strata they were likely to inhabit.
