# Supplementary material for: Habitat-based cetacean density models for the U.S. Atlantic and Gulf of Mexico
Source: Sci Rep. 2016 Mar 3;6:22615. doi: 10.1038/srep22615 (PMC4776172; doi:10.1038/srep22615)
Supplement: Supplementary Information [file srep22615-s1.pdf]

Habitat-based cetacean density models for the U.S. Atlantic and Gulf of Mexico

Supplementary Information

Jason J. Roberts, Benjamin D. Best, Laura Mannocci, Ei Fujioka, Patrick N. Halpin, Debra L. Palka, Lance P. Garrison, Keith D. Mullin, Timothy V. N. Cole, Christin B. Khan, William A. McLellan, D. Ann Pabst & Gwen G. Lockhart

Supplementary Methods

Sightings and Modeled Taxa. In the EC, we retained 21,946 cetacean sightings for analysis, omitted 4,786 sightings, and modeled 25 individual species and 3 multi-species guilds (Supplementary Tables S1, S2). In the GOM, we retained 4,361 sightings and omitted 585, and modeled 17 individual species and 2 multi-species guilds.

Detection Hierarchy. Buckland et al. recommended that at least 60-80 sightings be used to fit a detection function[1], but stated that the number of sightings required depends on the level of precision desired, and a simulation study found that low bias can be achieved for some detection functions with just 30 sightings, the fewest number tested in that study[2]. These results collectively indicate that there is no hard-and-fast minimum, but to maximize precision and minimize bias, one should avoid fitting detection functions with few sightings. When too few are available, a typical workaround is to pool sightings from multiple surveys or species, then apply the fitted detection function to all of them[3]. With this approach in mind, we arranged our surveys in two hierarchies—aerial and shipboard—that grouped them according to similarity of observation protocol and platform, and used the hierarchies to guide our pooling decisions.

For the aerial hierarchy (Supplementary Fig. S1), we first split off the North Atlantic Right Whale Sighting Surveys (NARWSS) from the rest. Its primary mission was to locate right whales, alert mariners of their presence, and obtain photographs for mark-recapture analysis[4]. Although NARWSS aircraft utilized bubble windows, NARWSS did not have a belly observer and the survey protocol required observers to dedicate less attention to the observing the area immediately below the aircraft than is recommended for distance sampling, necessitating special treatment during analysis (see below). We split up the remaining surveys according to whether a belly observer was used, the altitude flown, the surveyor organization, and the survey program. For the shipboard hierarchy (Supplementary Fig. S2), we first split off surveys performed with the naked eye from those performed with binoculars. We then split the binocular surveys first according to platform height, then vessel, surveyor organization, and survey region.

To ease the problem of obtaining sufficient sightings of rare species to fit detection functions we incorporated additional sightings from surveys conducted outside of our study area. These include the REMMOA and NOAA surveys of the Caribbean[5,6], the MAR-ECO survey of the mid-Atlantic ridge[7], and the SCANS II and CODA shipboard surveys of the European Atlantic[8]. We used these surveys only in fitting detection functions; we did not use them in the spatial modeling stage of the analysis.

Detection Functions. At each node of the detection hierarchies, we tallied the number of sightings of the modeled taxon reported by all surveys under that node. When a suitable number of sightings existed under a node, typically 60 or more, we fitted a detection function specific to those surveys. If too few were available, we ascended the hierarchy to the parent node and tried again. If we ascended very high in the hierarchy—typically to child nodes of the "all surveys" node at the top—without obtaining sufficient sightings, we pooled sightings of additional "proxy" species into that branch of the hierarchy and started over. For example, when modeling humpback whales, too few humpback sightings were obtained from shipboard surveys to fit humpback-specific detection functions, despite pooling many years of surveys. To compensate, we added sightings of all other baleen whales as proxies for

humpbacks, which allowed us to fit several shipboard detection functions. For proxy species, we consulted the literature and species experts for selecting species that displayed similar size, behaviors, and other characteristics that affect detectability. The taxon-specific supplementary reports (available at the OBIS-SEAMAP repository) specify, for each detection function, whether proxy species were used, which ones were used, and how many of each were sighted.

Following Buckland et al., before fitting each detection function we right-truncated the sightings at a distance that removed any "long tail" of distant sightings[1], typically resulting in a loss of roughly 5% of the sightings. We then fitted a number of formulations and selected the one with the lowest Akaike information criterion (AIC). We tested both conventional distance sampling (CDS) and multiple-covariate distance sampling (MCDS) formulations. For CDS, we tested hazard rate (HR) and half normal (HN) key functions with no adjustments, HR with second and forth order polynomial adjustments, HN with second and third order cosine adjustments, and HR with a forth order Hermite polynomial adjustment. For MCDS, we tested as covariates the group size (number of sighted animals), the Beaufort sea state, the observer's subjective estimate of the quality of observation conditions (or sun glare, if quality was not available), the survey ID (when sufficient sightings were reported by all surveys in the pool), and the vessel or aircraft that was used. When pooling multiple surveys, we only used covariates that were defined and collected the same way among all surveys in the pool (e.g., as occurred when pooling multiple surveys from the same surveyor organization). We discarded covariates that produced obvious erroneous effects (e.g., when detectability was predicted to increase with increasing Beaufort sea state).

Although large surveys such as NARWSS occasionally reported enough sightings to fit detection functions on a per-survey basis, exploratory analysis showed that per-survey detection functions fitted to a series of very similar surveys almost always achieved poorer fits than a single detection function fitted to all of them together. For this reason, we rarely fitted detection functions on a per-survey basis.

Several aerial survey programs measured vertical angles to sightings using marks on windows or wing struts, resulting in "heaping" of distance values[1], typically at 10° increments. For these, we fitted detection functions to the heaps, using angular cutpoints that were halfway between the heaped values[1]. Several aerial programs also suffered from an inadequate view of the survey trackline, due to not having a belly observer or bubble windows, or, in the case of the NARWSS program, to observers not focusing adequate attention on the trackline, resulting in missed detections at short distances. For these surveys, we applied left truncation[1].

We fitted all detection functions using the R *mrds* package version 2.1.10. The taxon-specific supplementary reports available at the OBIS-SEAMAP repository document the pooling that was done and the detection functions that were fitted, along with statistical diagnostics.

Probability of Detection along the Trackline. Distance sampling methodology assumes that the probability of detecting objects that lie along the trackline (i.e. at distance 0) is 1. This is often called the "$g(0)=1$" assumption. Unfortunately this assumption often does not hold for cetacean surveys. Cetaceans dive; while submerged, they are unavailable to be detected at the surface. Cetaceans may also be difficult for observers to perceive, due to their size, coloration, or failure to display obvious visual cues[9]. These two problems are known as *availability bias* and *perception bias* respectively and result in an underestimation of abundance unless they are accounted for[10].

A recommended solution is to utilize two independent observer teams and perform a mark-recapture distance sampling analysis[11]. This approach was closed to us, as most of our surveys used a single observer team. Instead, we consulted the literature to obtain estimates of $g(0)$ that incorporated these biases and then, when applying the detection functions to estimate abundance for each survey segment, we scaled the estimated abundance with the inverse of $g(0)$ [3]. The taxon-specific supplementary reports available at the OBIS-SEAMAP repository document the $g(0)$ estimates we used.

Splitting of Survey Transects into Segments. Concluding the first stage of the analysis, we split the survey transects into segments and predicted abundance for each segment. Prospective model users requested density surfaces with 10 km resolution, therefore we sought to obtain segments of this length. For each survey, we first iterated through the sequence of points that defined the transects, finding sections of continuous survey effort, defined as a sequence of effort points for which there were no off-effort gaps of 1 h or more and no stretch of 15 km for which 1/3 or more of it was off-effort. We then split each continuous section into equal-length on-effort segments, as follows.

First, we computed the number of segments for the continuous section, *n*, by dividing its length by 10 km using integer division. If the remainder was less than 5 km, we distributed the remainder evenly among the *n* segments, resulting in *n* segments slightly larger than 10 km. Otherwise, we increased the number of segments by 1 and computed their length by dividing the continuous section's length by *n*+1, resulting in *n*+1 segments slightly smaller than 10 km. Under no circumstances did a segment span more than 15 km of effort. A very small number of short, spatiotemporally-isolated segments occurred and were preserved, so long as they were longer than 1 km.

In the EC region, the segmenting procedure yielded 106,813 segments with a mean length of 9.95 km (SD=0.89 km). In the GOM, it yielded 19,988 segments of 9.75 km (SD=1.56 km).

Delineation of Seasonal and Sub-Regional Strata. Some cetacean species, particularly baleen whales, migrate between biogeographic regions as part of their reproductive cycle. Modeling the density of these species from environmental predictors can be problematic, as their environmental preferences may change between times of year—e.g. during summer, baleen whales might prefer cold, productive waters for feeding; during winter, they might prefer warm, calm waters far from predators for calving[12]. To address this, we reviewed what was known of the life history of each taxon. If the literature suggested the taxon exhibits seasonality in which its relationship to the environment is expected to be different during different times of year, we split the year into taxon-specific seasons to be modeled with separate spatial models (fitting each seasonal model to the segments from that season), provided that we had sufficient survey coverage and sightings to model at least one of the seasons effectively, and that the spatial pattern in the sightings resembled the expectation described by the literature. We delineated seasons at month boundaries. If the literature offered no conclusive description of seasonality or we lacked the data to reproduce it, we modeled the taxon with a single "year-round" spatial model.

After investigating seasonality and, when appropriate, splitting the segments into seasonal strata, we reviewed what was known about the spatial ecology of the taxon during each season. When the known ecology of the taxon indicated that it either exhibited ecologically different behaviors in different parts of the study area (Supplementary Fig. S3), was typically absent from an area (Supplementary Fig. S4), or there was reason to believe a taxon was present but we lacked the survey data to confidently model its density (Supplementary Fig. S3), we split the study area into sub-regional strata and modeled them separately. The taxon-specific supplementary reports available at the OBIS-SEAMAP repository document the seasonal and sub-regional strata we defined.

Spatial Models. For each taxon, after splitting the data into seasonal and sub-regional strata we fitted a generalized additive model (GAM) to the segments in each stratum, using abundance on the segment as the response variable, the natural logarithm of the surveyed area as the offset (calculated as $2(w_R-w_L)L$, where w_R is the right-truncation distance of the detection function, w_L is the left truncation distance (or 0 of left truncation was not applied) and L is the segment length), and environmental covariates believed to correlate with cetacean distributions (Supplementary Table S3). We only considered covariates that were appropriate for the ecology of the taxon and the sub-region of interest. For example, mesoscale eddies shed from the Gulf Stream or Gulf of Mexico Loop Current rarely maintain coherence over the continental shelf, so we only used "distance to eddy" covariates in models of off-shelf sub-regions.

We obtained all covariates from gridded remote sensing and ocean model products (Supplementary Table S3). After obtaining the original products, we projected them to a 10 km resolution grid that used an Albers equal area map projection designed to minimize spatial error within the study areas. Next, we prepared 8-day climatologies for the dynamic products by binning and averaging the available time series. Finally, we obtained covariate values for the survey segments by interpolating the values of the 10 km grids at the segment centroids. For physiographic covariates we used bilinear interpolation. For dynamic oceanographic covariates, we obtained contemporaneous and climatological values using trilinear interpolation, with either the date of the survey (for contemporaneous values) or the day of the year (for climatological values) as the time dimension. We performed all geoprocessing and sampling of covariates using ArcGIS 10.2.2 and the Marine Geospatial Ecology Tools software[13].

For strata having more than 40 sightings, we fitted multivariate models. For strata having 20-40 sightings, we fitted univariate models so as to be parsimonious and not risk overfitting the model. For some of these we tested many covariates and selected the one that explained the most deviance; for others, we selected a specific covariate based on the known ecology of the taxon. Finally, when less than 20 sightings were available, we fitted a model with no covariates, producing a mean density estimate for the modeled stratum; this is typically known as a "stratified model".

When a model included dynamic oceanographic covariates, we fitted three versions of it. The first used 8-day climatological estimates of the covariates (e.g. mean sea surface temperature (SST) for a given 8 days of the year, averaged over 30 years). These allowed the model to consider regular seasonal variations but not inter-annual and ephemeral variations. The second version used contemporaneous estimates (e.g. SST on the date of the survey segment, from a daily satellite image). These allowed the model to capture a full range of temporal variations—inter-annual, seasonal, and ephemeral—but suffered from data loss due to clouds or because satellites had not been aloft for the entire 1992-2014 study period. We restricted this version to the segments that had values for all covariates under consideration. The third version used climatological covariates but was restricted to the contemporaneous version's segments, to obtain a climatological version whose goodness-of-fit statistics were directly comparable to the contemporaneous version's statistics (by virtue of being fitted to the same segments). To distinguish between the first and third versions, which both used climatological covariates, we refer to them as the "climatological model" and "climatological-same-segments model", respectively.

To alleviate the data loss problem with contemporaneous covariates, we usually tested the three model versions with a sequence of candidate formulations that progressively added covariates derived from increasingly modern satellites, introducing more explanatory power at the cost of losing more survey segments. The first formulation included just physiographic covariates, resulting in no data loss. In this case, only one model version was necessary, as no dynamic covariates were used. The second formulation added SST and wind covariates. These data were available for the entire 1992-2014 study period, and we used products that blended observations from multiple satellites to fill data gaps, resulting in very little loss of survey data. The third formulation added covariates derived from ocean currents. These were also bended, gap-filled products but were only available for 1993-2013, forcing us to discard surveys from 1992 and 2014. The fourth formulation added biological covariates. Not all of these were gap-filled; we applied a Gaussian smoother that reduced the data gaps to < 10% of the pixels. These were only available from late 1997-2013 or 2014, depending on the covariate; we discarded surveys performed outside this window, resulting in a substantial loss of survey data, particularly in the GOM region.

We fitted all models using the R *mgcv* package version 1.8-4 [14,15]. We used thin-plate regression splines with shrinkage (`bs="ts"`). To help preserve the ecological interpretability of functional relationships, we limited each spline to 4 degrees of freedom. We used a shrinkage approach for selecting covariates for the models: after model fitting, if a covariate p-value was greater than 0.05 or its estimated degrees of freedom were less than 0.85 (resulting in its estimated confidence limits enclosing 0 throughout the range of the covariate), we removed the covariate from the model and refitted it. We assumed the Tweedie distribution and allowed *mgcv* to estimate the Tweedie p parameter (`family=tw()`). We used the REML optimization method[16]. The taxon-specific supplementary reports available at the OBIS-SEAMAP repository document the model formulations and provide diagnostic plots and statistics. During model checking procedures, we examined Q-Q plots of deviance residuals and plots of random quantile residuals vs. the linear predictor, among other diagnostics. We also produced correlograms of scaled Pearson residuals to check for autocorrelation. Autocorrelation occasionally occurred at a lag of 1 segment, but was generally low (e.g. $r < 0.05$) but statistically significant at the $p < 0.05$ level due to the large number of segments in the study. Generalized additive mixed models (GAMMs) have been recommended to address autocorrelation[17]; this was beyond the scope of our study but remains for consideration in a future revision of our models.

Prediction of Density Surfaces. For each of the three model versions, we selected the formulation that explained the most deviance and then predicted density surfaces from the fitted model applied to a 10 km grids of the environmental covariates. We predicted the climatological and climatological-same-segments models on each of the 8-day climatological periods spanned by the season, resulting in a maximum of 46 8-day predictions (for a year-round model). We predicted the contemporaneous model at a 1-day time step across the time period for which both survey data and covariates were available. For example, for Gulf of Mexico sperm whales, a year-round model was used, yielding 365 predictions per year (366 on leap years). Of the four model formulations tested, the model with physiographic, SST, and current covariates explained the most deviance. The covariates were available from 1993-2013, while the survey data were available from 1992-2009; therefore the prediction period was all days of 1993-2009, comprising 6209 daily predictions.

Selection of Best Models and Summarization of Density Surfaces. After predicting density surfaces for the three model versions, we inspected them and the model diagnostics, and selected either the climatological or contemporaneous version as our "best" model for the taxon. This was informed by the models' statistical performance, the spatiotemporal noisiness of the predictions, how much survey data was lost in the

contemporaneous model, and our judgment of how well the predictions matched well-established findings from the literature. All else being equal, we selected the model that explained the most deviance.

Prospective model users requested that predictions from the best model be summarized climatologically at a monthly time step—i.e., for each taxon, they wanted 12 density surfaces, one for each month, with each estimating the mean density of the taxon during that month, averaged over all years of the study. To confidently summarize the predictions at a monthly time step, we required: 1) evidence in the literature of the taxon shifting distribution seasonally, 2) sufficient survey coverage, both spatially and temporally, to detect the shift, and 3) a spatial pattern in the sightings and monthly-summarized predictions that resembled the expectation described by the literature. If all of these conditions were met, we produced monthly summaries for model users. If any were not, we produced a single seasonal summary that spanned all months of the season.

When there were so few sightings that we fitted a traditional stratified model, we applied the mean density estimate across the modeled season and sub-region, yielding a density surface that had the same value across the season and sub-region.

For comparison to other modeling efforts, such as those from the NMFS Stock Assessment Reports, we produced total abundance estimates for each season by computing the mean density of all pixels in the modeled area and multiplying by its area (main text Figs. 2-5, Supplementary Table S3).

Estimation of Uncertainty. In tandem with the density surfaces and total abundance predictions, we also produced uncertainty estimates using the method described by Miller et al.[17], Appendix B, section 3.2, adapted from the implementation of the `dsm.var.gam` function of the R *dsm* package version 2.2.5. For the seasonally or monthly-summarized density surfaces, we produced corresponding surfaces representing the standard error (SE) and coefficient of variation (CV) of each density surface pixel, applying Miller et al.'s method on a per-pixel basis. To estimate SE and CV for the total abundance predictions, we applied Miller et al.'s method to all of the pixels together.

The SE and CV estimates represented the uncertainty in the spatial model only (i.e. of the GAM parameter estimates, or of the mean density estimate when a stratified model was fitted), and therefore underestimate the true uncertainty. Traditionally, SE and CV estimates for density surface models incorporate the uncertainty in the detection functions and $g(0)$ estimates as well. Miller's method could not incorporate these sources of uncertainty when more than one detection function or $g(0)$ estimate was used, as occurred with all of our models. Barring new statistical innovations, the only way to incorporate these is to perform a nonparametric bootstrap. This was intractable with the computing resources available for our project; the models that used contemporaneous covariates required as many as 10^8 predictions per model for the EC region; properly bootstrapping these models would increase this to 10^{11} predictions. We plan to secure the necessary computational resources to accomplish this in a future revision of our models.

Supplementary Tables

Supplementary Table S1. Modeled taxa, with counts of sightings reported and retained in each analysis region. Notes: 1: We classified these ambiguous sightings as the modeled taxon from environmental, day of year, or group size covariates. 2: In the EC, we counted ambiguous "Bryde's or sei whale" sightings in both the Bryde's whale and sei whale models, as a precautionary measure. 3: In the GOM, we classified ambiguous "Bryde's or sei whale" and "Balaenoptera spp." sightings as Bryde's whales, following Maze-Foley and Mullin[18]. 4: We modeled these species as a guild; too few fully-resolved sightings were reported to build a classifier from them. 5: Only short-finned pilot whales occur in the GOM; we did not need the "pilot whales" guild there. 6: We lacked sufficient pantropical spotted dolphin sightings to fit a classification model for these ambiguous sightings; they all occurred in a northerly area near sightings of Atlantic spotted dolphins, so we treated them as such. 7: For harbor porpoises, we restricted the analysis to data collected when the Beaufort sea state was 2 or less, following Hammond et al.[19].

Group	Modeled taxon	Identification reported by observer	Sightings EC	Sightings GOM	Note
Small delphinoids	Atlantic spotted dolphin (*Stenella frontalis*)	Atlantic spotted dolphin	795	312	
		Atlantic spotted or bottlenose dolphin	33	35	1
		Atlantic or pantropical spotted dolphin	10		6
		Total:	838	347	
	Atlantic white-sided dolphin (*Lagenorhynchus acutus*)	Atlantic white-sided dolphin	1,670		
		Atlantic white-sided or short-beaked common dolphin	596		
		Total:	2,266		
	Bottlenose dolphin (*Tursiops truncatus*)	Bottlenose dolphin	4,603	1,733	
		Atlantic spotted or bottlenose dolphin	54	116	1
		Total:	4,657	1,849	
	Clymene dolphin (*Stenella clymene*)	Clymene dolphin	11	78	
	Fraser's dolphin (*Lagenodelphis hosei*)	Fraser's dolphin	2	5	
	Harbor porpoise (*Phocoena phocoena*)	Harbor porpoise	2,018		7
	Pantropical spotted dolphin (*Stenella attenuata*)	Pantropical spotted dolphin	17	719	
	Rough-toothed dolphin (*Steno bredanensis*)	Rough-toothed dolphin	11	51	
	Short-beaked common dolphin (*Delphinus delphis*)	Short-beaked common dolphin	938		
		Atlantic white-sided or short-beaked common dolphin	251		1
		Total:	1,189		
	Spinner dolphin (*Stenella longirostris*)	Spinner dolphin	2	71	
	Striped dolphin (*Stenella coeruleoalba*)	Striped dolphin	195	92	
	White-beaked dolphin (*Lagenorhynchus albirostris*)	White-beaked dolphin	12		

(continued next page)

Large delphinoids	False killer whale (*Pseudorca crassidens*)	False killer whale		2	19	
	Killer whale (*Orcinus orca*)	Killer whale		4	16	
	Melon-headed whale (*Peponocephala electra*)	Melon-headed whale		4	25	
		Melon-headed or pygmy killer whale			4	1
			Total:	4	29	
	Pilot whales (guild) (*Globicephala* spp.)	Long-finned pilot whale (*G. melas*)				
		Short-finned pilot whale (*G. macrorhynchus*)		86		
		Long-finned or short-finned pilot whale		823		
			Total:	909		4
	Pygmy killer whale (*Feresa attenuata*)	Pygmy killer whale			18	
		Melon-headed or pygmy killer whale			9	1
			Total:		27	
	Risso's dolphin (*Grampus griseus*)	Risso's dolphin		721	282	
	Short-finned pilot whale (*Globicephala macrorhynchus*)	Short-finned pilot whale			50	5
Beaked and sperm whales	Beaked whales (guild) (*Mesoplodon* and *Ziphius* spp.)	Blainville's beaked whale (*M. densirostris*)		3	2	
		Cuvier's beaked whale (*Z. cavirostris*)		46	22	
		Gervais' beaked whale (*M. europaeus*)		3	1	
		Sowerby's beaked whale (*M. bidens*)		14		
		True's beaked whale (*M. mirus*)		3		
		Unidentified *Mesoplodon* spp.		137	42	
		Unidentified *Mesoplodon* spp. or *Ziphius* sp.		20	49	
			Total:	226	116	4
	Kogia whales (guild) (*Kogia* spp.)	Dwarf sperm whale (*K. sima*)		4	16	
		Pygmy sperm whale (*K. breviceps*)		3	41	
		Dwarf or pygmy sperm whale		24	167	
			Total:	31	219	4
	Northern bottlenose whale (*Hyperoodon ampullatus*)	Northern bottlenose whale		4		
	Sperm whale (*Physeter macrocephalus*)	Sperm whale		501	360	

(continued next page)

Baleen whales	Blue whale (*Balaenoptera musculus*)	Blue whale	8		
	Bryde’s whale (*Balaenoptera edeni*)	Bryde’s whale		17	
		Bryde’s or sei whale	4	4	2,3
		Unidentified *Balaenoptera* spp.		4	3
		Total:	4	25	
	Fin whale (*Balaenoptera physalus*)	Fin whale	1,690	1	
		Fin or sei whale	410		
		Total:	2,100	1	
	Humpback whale (*Megaptera novaeangliae*)	Humpback whale	2,732		
	Minke whale (*Balaenoptera acutorostrata*)	Minke whale	1,031		
	North Atlantic right whale (*Eubalaena glacialis*)	North Atlantic right whale	1,634		
	Sei whale (*Balaenoptera borealis*)	Sei whale	585		
		Bryde’s or sei whale	4		2
		Fin or sei whale	232		1
		Total:	821		
		Grand total, all sightings:	21,946	4,361	

Supplementary Table S2. Ambiguous sightings omitted from the analysis. For “Bottlenose or rough-toothed dolphin”, we lacked the sightings necessary to attempt a habitat-based classification. For “Risso's or Bottlenose dolphin”, the sighting occurred in habitat occupied by both species, making habitat-based classification inconclusive. For the rest, the reported identifications were too generic for classification.

Identification reported by observer	EC	GOM
Bottlenose or rough-toothed dolphin	1	
Common dolphin or *Stenella* spp.	1	
Risso's or Bottlenose dolphin	1	
Unidentified *Balaenoptera* spp.	21	
Unidentified baleen whale	18	
Unidentified cetacean	37	
Unidentified delphinid	1,020	335
Unidentified dolphin	6	
Unidentified large whale	796	38
Unidentified medium whale	19	
Unidentified odontocete	2,408	118
Unidentified porpoise or dolphin	2	
Unidentified small cetacean	3	
Unidentified small delphinid	5	
Unidentified small whale	52	44
Unidentified *Stenella* spp.	24	50
Unidentified whale	372	
Total:	4,786	585

Supplementary Table S3. Candidate covariates for the spatial models. All covariates were rescaled to the 10 km Albers equal area map projection used for the analysis. Each model only considered the covariates that were appropriate for the modeled region and known ecology of the taxon.

Type	Covariates	Resolution	Time range	Description
Physiographic	Depth, Slope	30 arc sec		Seafloor depth and slope, derived from SRTM30-PLUS global bathymetry[20]
	DistToShore, DistTo125m, DistTo300m, DistTo1500m	30 arc sec		Distance to the closest shoreline, excluding Bermuda and Sable Island, and various ecologically-relevant isobaths[20]
	DistToCanyon, DistToCanyon OrSeamount	30 arc sec		Distance to the closest submarine canyon, and to the closest canyon or seamount[21]
SST & Winds	SST, DistToFront	0.2°, daily	1991-2014	Foundation sea surface temperature (SST), from GHRSST Level 4 CMC SST[22], and distance to the closest SST front identified with the Canny edge detection algorithm[23]
	WindSpeed	0.25°, daily	1991-2014	30-day running mean of NOAA NCDC 1/4° Blended Sea Winds[24]
Currents	TKE, EKE	0.25°, daily	1993-2013	Total kinetic energy (TKE) and eddy kinetic energy (EKE), from Aviso 1/4° DT-MADT geostrophic currents
	DistToEddy, DistToAEddy, DistToCEddy	0.25°, weekly	1993-2013	Distance to the ring of the closest geostrophic eddy having any (DistToEddy), anticyclonic (DistToAEddy), or cyclonic (DistToCEddy) polarity, from Aviso 1/4° DT-MADT using a revision of the Chelton et al. algorithm[25]; we tested eddies at least 9, 4, and 0 weeks old
Biological	Chl	9 km, daily	1997-2014	GSM merged SeaWiFS/Aqua/MERIS/VIIRS chlorophyll (Chl) *a* concentration[26], smoothed with a 3D Gaussian smoother to reduce data loss to < 10%
	VGPM, CumVGPM45, CumVGPM90	9 km, 8 days	1997-2014	Net primary production (mg C m^{-2} day^{-1}) derived from SeaWiFS and Aqua using the Vertically Generalized Production Model (VPGM)[27]; we tested the original 8 day estimates as well as 45 and 90 day running accumulations
	PkPP, PkPB	0.25°, weekly	1997-2013	Zooplankton production (PkPP; g m^{-2} day^{-1}) and biomass (PkPB; g m^{-2}) from the SEAPODYM ocean model[28]
	EpiMnkPP, EpiMnkPB	0.25°, weekly	1997-2013	Epipelagic micronekton production (EpiMnkPP; g m^{-2} day^{-1}) and biomass (EpiMnkPB; g m^{-2}) from the SEAPODYM model[28]

Supplementary Table S4. Summary of results, by modeled taxon, region, and season. Model type "both" means one sub-region was modeled with a DSM and another was modeled with a stratified model. Prediction resolution gives the temporal resolution of the density surface predicted by the best model. % Area covered, $\hat{N}$, and CV give the percentage of the study area covered by the surface, total abundance of the surface averaged across the season, and coefficient of variation (CV) of the total abundance. The CV estimate only incorporates the uncertainty of the spatial model.

Group	Modeled taxon	Region	Season	Model type	Prediction resolution	% Area covered	$\hat{N}$	CV
Small delphinoids	Atlantic spotted dolphin	EC	Year-round	DSM	Year-round	100	55,436	0.32
		GOM	Year-round	DSM	Year-round	100	47,488	0.13
	Atlantic white-sided dolphin	EC	Year-round	DSM	Monthly	100	37,180	0.07
	Bottlenose dolphin	EC	Year-round	DSM	Monthly	100	97,476	0.06
		GOM	Year-round	DSM	Year-round	100	138,602	0.06
	Clymene dolphin	EC	Year-round	Stratified	Year-round	100	12,515	0.56
		GOM	Year-round	DSM	Year-round	100	11,000	0.16
	Fraser's dolphin	EC	Year-round	Stratified	Year-round	100	492	0.76
		GOM	Year-round	Stratified	Year-round	100	1,665	0.73
	Harbor porpoise	EC	Winter (Nov-May)	DSM	Monthly	56	17,651	0.17
			Summer (Jun-Oct)	DSM	Monthly	92	45,089	0.12
	Pantropical spotted dolphin	EC	Year-round	Stratified	Year-round	100	4,436	0.33
		GOM	Year-round	DSM	Year-round	100	84,014	0.06
	Rough-toothed dolphin	EC	Year-round	Stratified	Year-round	100	532	0.36
		GOM	Year-round	DSM	Year-round	100	4,853	0.19
	Short-beaked common dolphin	EC	Year-round	DSM	Monthly	100	86,098	0.12
	Spinner dolphin	EC	Year-round	Stratified	Year-round	100	262	0.93
		GOM	Year-round	DSM	Year-round	100	13,485	0.24
	Striped dolphin	EC	Year-round	DSM	Year-round	100	75,657	0.21
		GOM	Year-round	DSM	Year-round	100	4,914	0.17
	White-beaked dolphin	EC	Year-round	Stratified	Year-round	100	39	0.42

(continued next page)

Large delphinoids	False killer whale	EC	Year-round	Stratified	Year-round	100	95	0.84
		GOM	Year-round	Stratified	Year-round	100	3,204	0.36
	Killer whale	EC	Year-round	Stratified	Year-round	100	11	0.82
		GOM	Year-round	DSM	Year-round	100	185	0.41
	Melon-headed whale	EC	Year-round	Stratified	Year-round	100	1,175	0.50
		GOM	Year-round	DSM	Year-round	100	6,733	0.30
	Pilot whales (guild)	EC	Year-round	DSM	Year-round	100	18,977	0.11
	Pygmy killer whale	GOM	Year-round	DSM	Year-round	100	2,126	0.30
	Risso's dolphin	EC	Year-round	DSM	Monthly	100	7,732	0.09
		GOM	Year-round	DSM	Year-round	100	3,137	0.10
	Short-finned pilot whales	GOM	Year-round	DSM	Year-round	100	1,981	0.18
Beaked and sperm whales	Beaked whales (guild)	EC	Year-round	Both	Year-round	100	14,491	0.17
		GOM	Year-round	DSM	Year-round	100	2,910	0.16
	Kogia whales (guild)	EC	Year-round	Stratified	Year-round	100	678	0.23
		GOM	Year-round	DSM	Year-round	100	2,234	0.19
	Northern bottlenose whale	EC	Year-round	Stratified	Year-round	85	90	0.63
	Sperm whale	EC	Year-round	DSM	Monthly	100	5,353	0.12
		GOM	Year-round	DSM	Year-round	100	2,128	0.08
Baleen whales	Blue whale	EC	Year-round	Stratified	Year-round	100	11	0.41
	Bryde's whale	EC	Year-round	Stratified	Year-round	100	7	0.58
		GOM	Year-round	DSM	Year-round	100	44	0.27
	Fin whale	EC	Year-round	DSM	Monthly	100	4,633	0.08
		GOM	Year-round	Stratified	Year-round	100	9	1.01
	Humpback whale	EC	Winter (Dec-Mar)	DSM	Monthly	100	205	0.16
			Summer (Apr-Nov)	DSM	Monthly	100	1,637	0.07
	Minke whale	EC	Winter (Nov-Mar)	Both	Monthly	54	740	0.23
			Summer (Apr-Oct)	Both	Monthly	100	2,112	0.05
	North Atlantic right whale	EC	Winter (Nov-Feb)	DSM	Monthly	80	535	0.45
			Spring (Mar-Apr)	DSM	Monthly	78	416	0.12
			Summer (May-Jul)	Both	Monthly	80	379	0.07
			Fall (Aug-Oct)	DSM	Monthly	93	334	0.25
	Sei whale	EC	Winter (Dec-Mar)	Stratified	Monthly	79	98	0.25
			Spring (Apr-Jun)	DSM	Monthly	80	627	0.14
			Summer (Jul-Sep)	DSM	Monthly	100	717	0.30
			Fall (Oct-Nov)	DSM	Monthly	11	37	0.19

Supplementary Figures

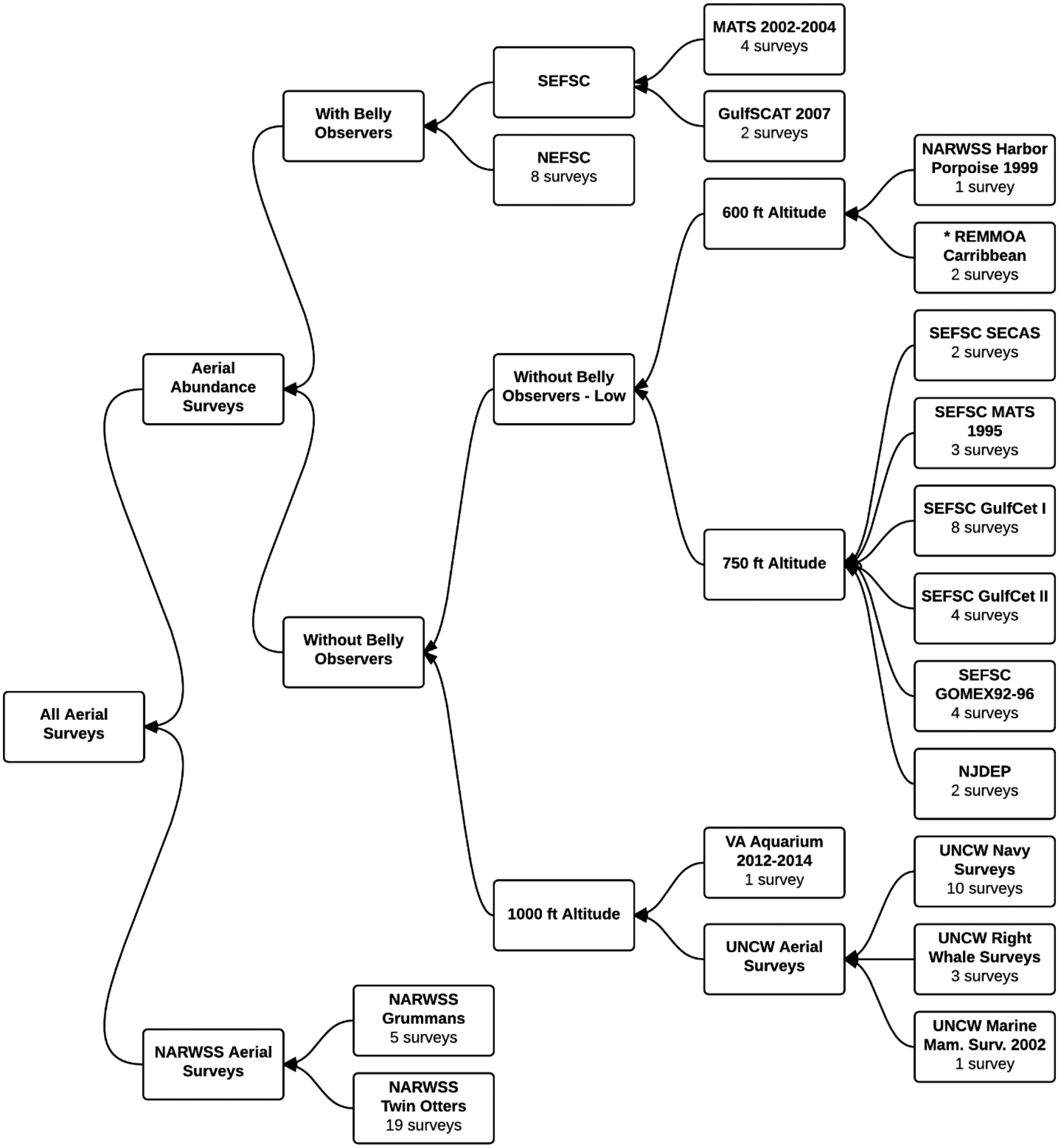

Supplementary Figure S1. Detection hierarchy for aerial surveys. Surveys marked with * were used to fit detection functions but not the spatial model.

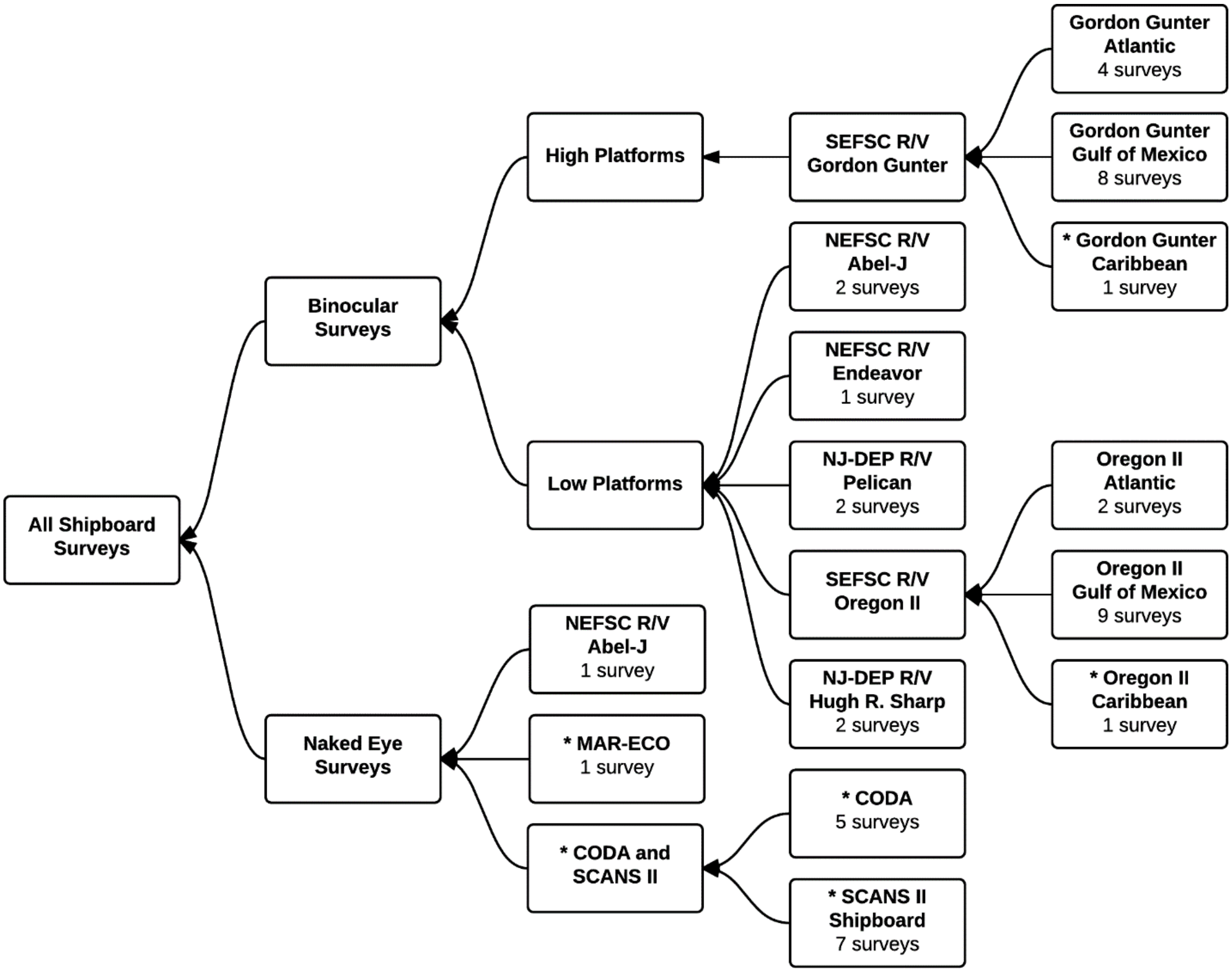

Supplementary Figure S2. Detection hierarchy for shipboard surveys. Surveys marked with * were used to fit detection functions but not the spatial model.

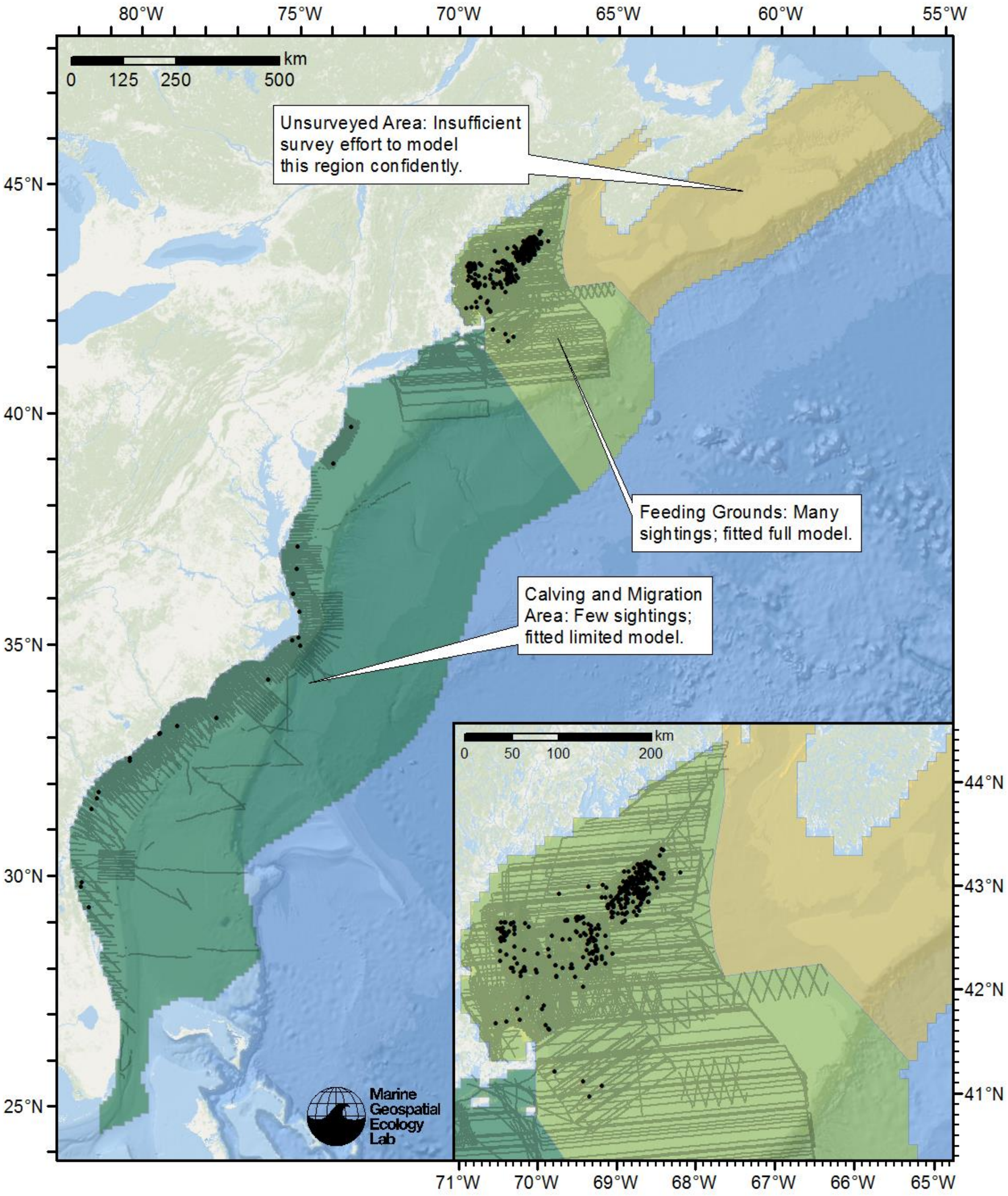

Supplementary Figure S3. Schematic for the EC North Atlantic right whale winter season (November-February) model, showing an example in which we split the study area on the basis of sub-units of the population likely exhibiting different relationships to the environment (right whales overwintering on the feeding grounds vs. those on the calving grounds). This model also shows an example of where we suspected a taxon was present—Canadian waters, in this case—but lacked the survey effort to model it confidently. Figure produced with ArcGIS 10.2.2 (http://www.arcgis.com); background map credits: Esri, DeLorme, GEBCO, NOAA NGDC, and other contributors.

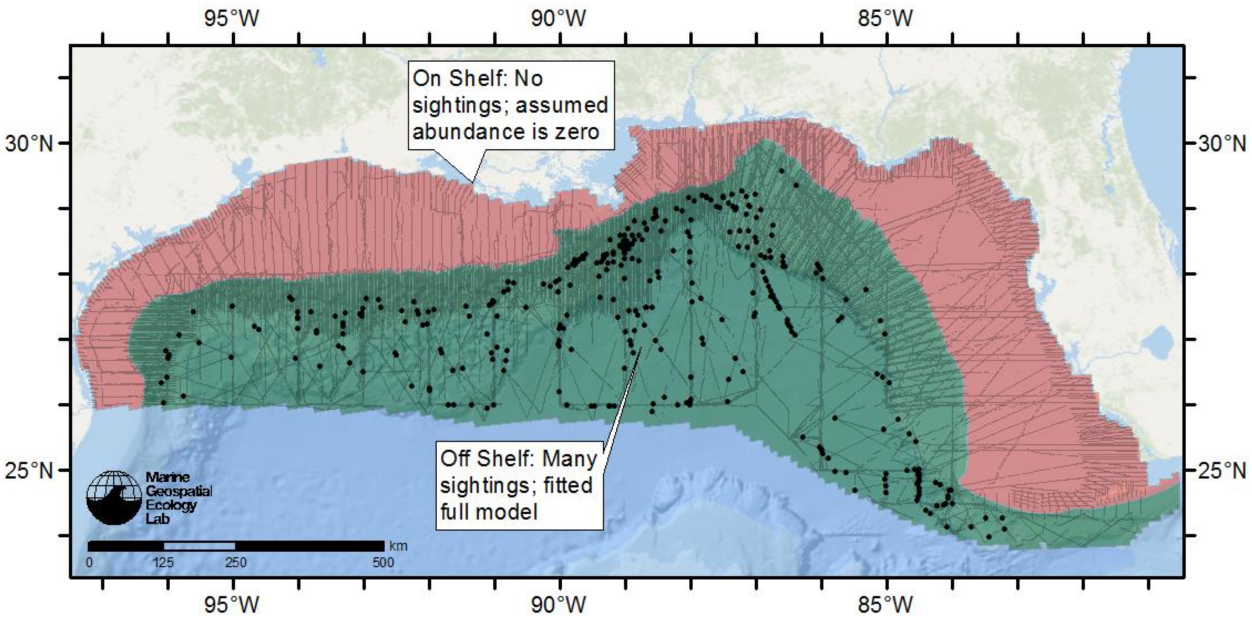

Supplementary Figure S4. Schematic for the GOM sperm whale year-round model, showing an example in which we split the study area on the basis of the taxon not occupying part of the area—the continental shelf, in this case. Figure produced with ArcGIS 10.2.2 (http://www.arcgis.com); background map credits: Esri, DeLorme, GEBCO, NOAA NGDC, and other contributors.

References

1. Buckland, S. T. *et al. Introduction to Distance Sampling: Estimating Abundance of Biological Populations*. (Oxford University Press, 2001).

2. Miller, D. L. & Thomas, L. Mixture models for distance sampling detection functions. *PloS One* **10,** e0118726 (2015).

3. Barlow, J. & Forney, K. A. Abundance and population density of cetaceans in the California Current ecosystem. *Fish. Bull.* **105,** 509–526 (2007).

4. Cole, T., Gerrior, P. & Merrick, R. L. *Methodologies of the NOAA National Marine Fisheries Service Aerial Survey Program for Right Whales (Eubalaena glacialis) in the Northeast U.S., 1998-2006*. (2007) Available at: http://www.nefsc.noaa.gov/publications/crd/crd0702/ (Accessed: 30th May 2014).

5. Mannocci, L. *et al.* Megavertebrate communities from two contrasting ecosystems in the western tropical Atlantic. *J. Mar. Syst.* **111-112,** 208–222 (2013).

6. Swartz, S. L. *et al. Visual and acoustic survey of humpback whales (Megaptera novaeangliae) in the Eastern and Southern Caribbean Sea: Preliminary Findings*. (2001) Available at: http://swfsc.noaa.gov/publications/CR/2001/2001Swartz.pdf (Accessed: 8th March 2014).

7. Waring, G. T., Nøttestad, L., Olsen, E., Skov, H. & Vikingsson, G. Distribution and density estimates of cetaceans along the mid-Atlantic Ridge during summer 2004. *J. Cetacean Res. Manag.* **10,** 137–146 (2008).

8. Hammond, P. S. *et al.* Cetacean abundance and distribution in European Atlantic shelf waters to inform conservation and management. *Biol. Conserv.* **164,** 107–122 (2013).

9. Kraus, S. D., Gilbert, J. R. & Prescott, J. H. A comparison of aerial, shipboard, and land-based survey methodology for the harbor porpoise, Phocoena phocoena. *Fish. Bull.* **81,** 910–913 (1983).

10. Thomas, L. *et al.* Distance Sampling in *Encyclopedia of Environmetrics 2nd edn* (John Wiley & Sons, 2013).

11. Burt, M. L., Borchers, D. L., Jenkins, K. J. & Marques, T. A. Using mark-recapture distance sampling methods on line transect surveys. *Methods Ecol. Evol.* **5,** 1180–1191 (2014).

12. Corkeron, P. J. & Connor, R. C. Why do baleen whales migrate? *Mar. Mammal Sci.* **15,** 1228–1245 (1999).

13. Roberts, J. J., Best, B. D., Dunn, D. C., Treml, E. A. & Halpin, P. N. Marine Geospatial Ecology Tools: An integrated framework for ecological geoprocessing with ArcGIS, Python, R, MATLAB, and C++. *Environ. Model. Softw.* **25,** 1197–1207 (2010).

14. Wood, S. N. & Augustin, N. H. GAMs with integrated model selection using penalized regression splines and applications to environmental modelling. *Ecol. Model.* **157,** 157–177 (2002).

15. Wood, S. *Generalized additive models: an introduction with R*. (CRC press, 2006).

16. Wood, S. N. Fast stable restricted maximum likelihood and marginal likelihood estimation of semiparametric generalized linear models. *J. R. Stat. Soc. Ser. B Stat. Methodol.* **73,** 3–36 (2011).

17. Miller, D. L., Burt, M. L., Rexstad, E. A. & Thomas, L. Spatial models for distance sampling data: recent developments and future directions. *Methods Ecol. Evol.* **4,** 1001–1010 (2013).

18. Maze-Foley, K. & Mullin, K. D. Cetaceans of the oceanic northern Gulf of Mexico: Distributions, group sizes and interspecific associations. *J. Cetacean Res. Manag.* **8,** 203–213 (2006).

19. Hammond, P. s. *et al.* Abundance of harbour porpoise and other cetaceans in the North Sea and adjacent waters. *J. Appl. Ecol.* **39,** 361–376 (2002).

20. Becker, J. J. *et al.* Global Bathymetry and Elevation Data at 30 Arc Seconds Resolution: SRTM30_PLUS. *Mar. Geod.* **32,** 355–371 (2009).

21. Harris, P. T., Macmillan-Lawler, M., Rupp, J. & Baker, E. K. Geomorphology of the oceans. *Mar. Geol.* **352,** 4–24 (2014).

22. Brasnett, B. The impact of satellite retrievals in a global sea-surface-temperature analysis. *Q. J. R. Meteorol. Soc.* **134,** 1745–1760 (2008).

23. Canny, J. F. A computational approach to edge detection. *IEEE Trans. Pattern Anal. Mach. Intell.* **8,** 679–698 (1986).

24. Zhang, H.-M., Bates, J. J. & Reynolds, R. W. Assessment of composite global sampling: Sea surface wind speed. *Geophys. Res. Lett.* **33,** (2006).

25. Chelton, D. B., Schlax, M. G. & Samelson, R. M. Global observations of nonlinear mesoscale eddies. *Prog. Oceanogr.* **91,** 167–216 (2011).

26. Maritorena, S., d'Andon, O. H. F., Mangin, A. & Siegel, D. A. Merged satellite ocean color data products using a bio-optical model: Characteristics, benefits and issues. *Remote Sens. Environ.* **114,** 1791–1804 (2010).

27. Behrenfeld, M. J. & Falkowski, P. G. Photosynthetic rates derived from satellite-based chlorophyll concentration. *Limnol. Oceanogr.* **42,** 1–20 (1997).

28. Lehodey, P., Murtugudde, R. & Senina, I. Bridging the gap from ocean models to population dynamics of large marine predators: a model of mid-trophic functional groups. *Prog. Oceanogr.* **84,** 69–84 (2010).
